# Immunohistological Characterization of Actinic Keratoses with Varying Degrees of Proliferation

**DOI:** 10.3390/cancers18091340

**Published:** 2026-04-23

**Authors:** Vasileios Dervenis, Conrad Falkenberg, Alexandra Knebel, Lutz Schmitz, Thomas Dirschka

**Affiliations:** 1 Department of Dermatology, Venereology and Allergology, St. Josef Hospital, Ruhr University Bochum, Gudrunstrasse 56, 44791 Bochum, Germany; l.schmitz@centroderm.de; 2 CentroDerm Clinic, Heinz-Fangman-Straße 57, 42287 Wuppertal, Germany; c.falkenberg@centroderm.de (C.F.); t.dirschka@centroderm.de (T.D.); 3 Department of OB/GYN and REI (UniKiD), Medical Faculty and University Hospital Duesseldorf, Heinrich Heine University Duesseldorf, 40255 Duesseldorf, Germany

**Keywords:** actinic keratosis, cutaneous squamous cell carcinoma, disease progression, basal proliferation, Ki-67, podoplanin, p53, p16

## Abstract

**Background:** Actinic keratoses (AKs) are considered early in situ forms of cutaneous squamous cell carcinoma (cSCC). However reliable histopathological or molecular markers for predicting the risk of progression are still lacking. The aim of this study was to investigate the relationship between immunohistochemical markers and basal proliferation patterns of AKs in order to identify histopathological associations that may be relevant for malignant transformation. **Methods:** A total of 97 AK samples from facial and scalp areas were retrospectively analyzed and classified according to their basal proliferation pattern (Pro I: non-proliferative and Pro III: proliferative). Immunohistochemical staining was performed for Ki-67, p53, p16 and podoplanin. In addition, histopathological parameters such as Röwert-Huber grade, inflammatory infiltrate, parakeratosis, elastosis and the presence of acantholysis were evaluated. **Results:** Pro III lesions were significantly more frequently associated with higher Röwert-Huber grades (AK III: 47.9% vs. 14.3%; *p* = 0.0004) and with acantholysis (*p* = 0.0004). No significant differences between the groups were found for Ki-67, p53 and p16. Podoplanin expression, however, was significantly higher in Pro III lesions (93.7% vs. 57.1%, *p* < 0.0001) and was predominantly localized basally. The combination of a PRO III pattern and podoplanin positivity identified a distinct histopathological subgroup associated with features linked to progression. **Conclusions:** Podoplanin expression, especially in combination with PRO III pattern and acantholysis, characterizes a histologically and biologically distinct AK subgroup. In contrast, Ki-67, p53 and p16 showed no additional discriminative value in this cohort. Podoplanin could therefore be a useful addition to existing classification systems and in the future support risk-adapted treatment decision. However, prospective longitudinal studies are required to determine its prognostic value.

## Simple Summary

Actinic keratoses (AKs) are sun-induced lesions that can progress to cutaneous squamous cell carcinoma, but the tools to identify higher-risk AKs are limited. We retrospectively analyzed 97 facial/scalp AK biopsies and grouped them by basal proliferation pattern (PRO I vs. PRO III). PRO III lesions were more often high-grade (AK III) and showed acantholysis. Ki-67, p53 and p16 staining did not distinguish proliferative from non-proliferative AKs. In contrast, podoplanin was frequently expressed in PRO III AKs (93.7% vs. 57.1%), predominantly localized to the basal layers. The combination of a PRO III pattern with podoplanin positivity (often with acantholysis) identifies a distinct histopathological subgroup associated with features previously linked to progression and may be relevant for future risk stratification and management.

## 1. Introduction

Actinic keratoses (AKs) are regarded as early in situ forms of cutaneous squamous cell carcinoma (cSCC) and arise through the proliferation of atypical keratinocytes within the epidermis [1]. AKs are a chronic condition that typically develops as a result of long-term exposure to ultraviolet (UV) radiation, occurring predominantly in continuously sun-exposed areas such as the face, bald scalp, forearms and the backs of the hands [2]. The frequency of AKs is particularly high in fair-skinned populations and increases significantly with age. Other risk factors include male sex, immunosuppression and certain environmental factors [3,4]. In industrialized nations, a continuous rise in incidence has been observed for decades, a trend attributed to demographic changes and altered leisure habits with increased sun exposure [5].

The risk of progression of an individual lesion into invasive cSCC varies widely in the literature, ranging from 0.025% to 16% per year [6]. This broad range, along with the lack of reliable clinical–histopathological risk factors and the potential danger of metastasis of cSCC, makes treatment of all AKs necessary. However, this leads to a risk of overtreatment, which burdens patients and has substantial economic consequences. For risk assessment, histopathological classifications are becoming increasingly important. The established Röwert-Huber [7] system (AK I–III) describes the degree of atypical keratinocytes but does not allow a reliable prediction of progression to invasive SCC (iSCC). Notably, AK I lesions are frequently observed in close proximity to iSCC lesions [8]. The PRO classification (PRO I–III) [9], which evaluates basal downward growth, represents a promising approach. A pronounced basal proliferation pattern (PRO III) and acantholysis may be associated with a higher progression risk and altered therapeutic response [10].

Against this background, the present study aimed to systematically analyze the histopathological characteristics of AKs and to combine them with immunohistochemical findings in order to explore histopathological and immunohistochemical associations that may be relevant to progression into invasive squamous cell carcinoma. Improved characterization of lesions with potentially more aggressive histopathological features may help refine future risk stratification approaches while avoiding unnecessary therapies.

## 2. Materials and Methods

### 2.1. Study Population

This retrospective, single-center analysis was conducted at the CentroDerm Clinic (Wuppertal, Germany) using the clinic’s internal database between January 2018 and June 2021. The study was performed in accordance with the Declaration of Helsinki (2013) and approved by the Ethics Committee of the University of Witten/Herdecke (No. S-135/2021). Biopsies were taken for various clinical indications, including confirmation of diagnosis, exclusion of iSCC, or treatment of individual lesions. A total of 100 tissue samples of AKs from the face and scalp were initially screened. Overall, three samples were excluded before final analysis, including two with insufficient histological quality for reliable evaluation and one in which histopathological reassessment revealed Bowen’s disease as a discrepant diagnosis. Samples were classified into two groups based on their basal proliferation pattern: Pro I (non-proliferative, *N* = 49) and Pro III (proliferative, *N* = 48). Lesions with a PRO II growth pattern were excluded a priori because the aim of the study was to compare two clearly distinct basal proliferation phenotypes. As PRO II represents an intermediate pattern, its inclusion would likely have reduced group separation and complicated the interpretation of potential immunohistochemical differences between the study groups. To ensure sample homogeneity, only AKs from the same anatomical regions (face and scalp) were included in the analysis. AKs located outside the head region were excluded. Immunohistochemical staining for p53, p16, Ki-67 and podoplanin was performed. Additionally, patient age and sex were recorded. The two groups were strictly separated and no patient was included in both.

### 2.2. Microscopic Evaluation

All histological sections (4 μm) stained with hematoxylin and eosin (H&E), as well as immunohistochemically with Ki-67, p53, p16 and podoplanin, were examined microscopically. Only specimens with preserved epidermis and dermis were included.

Basal proliferation patterns were classified according to Schmitz et al. [9]. PRO I (known as “crowding”) describes the clustering of basal atypical keratinocytes. PRO II (known as “budding”) is characterized by small buds of atypical keratinocytes protruding into the upper dermis but not exceeding the epidermal thickness. PRO III (“papillary sprouting”) presents pointed or filiform proliferations extending into the dermis, exceeding the epidermal thickness, but without evidence of invasive growth. All samples were also classified according to Röwert-Huber et al. [7]. In grade AK I, the atypical keratinocytes are confined to the lower third of the epidermis, in grade AK II, the atypical keratinocytes can be detected in the lower two-thirds of the epidermis, and in AK III, the entire epidermis is interspersed with atypical keratinocytes.

Quantitative analysis of Ki-67, p53, p16 and podoplanin expression was performed after digitalization of slides using a NanoZoomer® scanner (Hamamatsu Photonics K.K., Hamamatsu City, Shizuoka, Japan), followed by evaluation in PathoZoom® Digital Lab (Smart In Media AG, Cologne, Germany). Positive cells were identified and labeled and the results were then manually reviewed and adjusted.

For Ki-67, the proportion of immunoreactive nuclei (<1%, 1–20%, 21–40%, >41%), as well as their epidermal localization (lower third, lower two-thirds, entire epidermis), was recorded. P53 expression was semi-quantitatively assessed according to the percentage of positive nuclei (<1%, 1–25%, 26–50%, >51%), staining intensity (none, weak, moderate, strong) and epidermal localization, following Röwert-Huber et al. [7]. Additionally, the distribution of positive cells within or outside proliferative zones was documented. For p16, both the percentage of positive cells (<1%, 1–30%, >31%) and staining intensity (none, weak, moderate, strong) were considered. Nuclear and cytoplasmic signals were classified as positive, with additional assessment of epidermal localization. Evaluation of podoplanin expression was based on the semi-quantitative immunoreactive score (IRS) according to Remmele and Stegner [11,12]. The score was calculated as the product of the percentage of positive cells (0–4) and staining intensity (0–3). The proportion of immunoreactive cells was divided into five groups: 0 = no positive cells, 1 = 1–10%, 2 = 11–50%, 3 = 51–80%, 4 ≥ 81%. The staining intensity was rated on a four-point scale: 0 = none, 1 = weak, 2 = moderate, 3 = strong. This resulted in a total score of 0 to 12 points, which was interpreted as follows: 0 points = no expression, 1–2 points = weak, 3–4 points = moderate, 6–12 points = strong. Multiplying these scores together can practically never result in 5 points.

As no standardized scoring approach is currently available for the immunohistochemical assessment of these markers, the scoring categories were adapted to the distribution of our cohort in order to enable comparison both within our study and with the existing literature. For podoplanin, we applied a semiquantitative IRS-based categorization in line with previously published semiquantitative approaches. This was intended to allow a structured comparison within our cohort and should not be interpreted as a clinically validated threshold for defining podoplanin positivity or negativity.

Histological parameters (parakeratosis, elastosis, infiltrate) were semi-quantitatively evaluated (none, mild, moderate, strong) and acantholysis was recorded dichotomously, if present or not.

### 2.3. Statistical Analysis

Data analysis was performed using MedCalc Software Version 19.1.7 (MedCalc Software, Ostend, Belgium). The distribution of data was tested using the D’Agostino–Pearson test. Normally distributed data were expressed as mean and standard deviation (SD), otherwise they were expressed as median and interquartile range (IQR). Non-parametric data were analyzed using the Mann–Whitney U test for independent samples. *p* values less than 0.05 were considered statistically significant.

## 3. Results

A total of 97 AKs were included in this study (49 PRO I, 48 PRO III). The median age was 77 years (IQR 66–82 years) and the majority of patients were males (*n* = 66, 68%). Pro I lesions were significantly more often located on the scalp (38.8% vs. 14.6%; *p* = 0.0075), while Pro III lesions predominantly occurred on the forehead (39.6% vs. 18.4%; *p* = 0.0218).

Histologically, according to Röwert-Huber [7], AK I was more frequent in Pro I (40.8% vs. 18.8%; *p* = 0.0182), while AK III predominated in Pro III (47.9% vs. 14.3%; *p* = 0.0004), and AK II showed no significant difference between the Pro I and Pro III groups (*p* = 0.2458).

Parakeratosis and elastosis showed no significant differences between the comparison groups (*p* = 0.0588 and *p* = 0.2600, respectively). An inflammatory infiltrate was significantly less frequent in Pro I (44.9% vs. 6.3%; *p* < 0.0001), whereas moderate intensity of infiltrate predominated in Pro III (29.2% vs. 6.1%; *p* = 0.0030). Acantholysis occurred exclusively in Pro III (11/97; *p* = 0.0004). Demographic and histological characteristics are summarized in Table 1.

**Figure 1:**
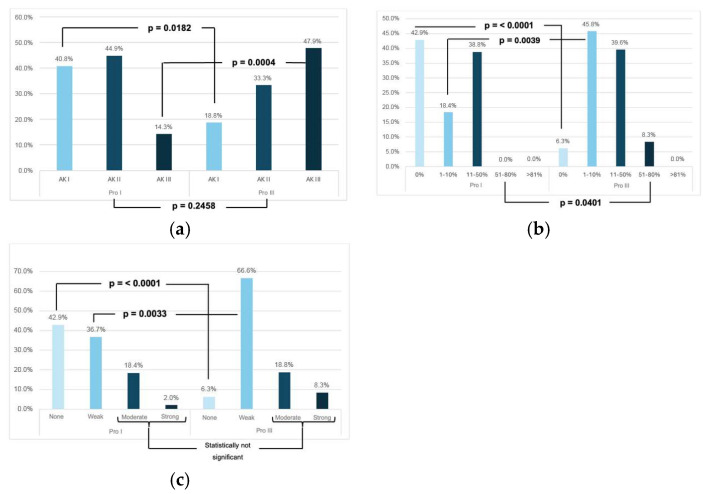
Comparison of histological features and podoplanin immunoreactivity between non-proliferative (Pro I) and proliferative (Pro III) actinic keratoses. (**a**) Distribution of histological grades (AK I–III) within both groups. Low-grade lesions (AK I) were significantly more common in the non-proliferative group (Pro I), whereas high-grade lesions (AK III) were significantly more frequent in the proliferative group (Pro III). (**b**) The proportion of podoplanin-positive cells was categorized into five groups (0%, 1–10%, 11–50%, 51–80%, >81%). Podoplanin expression was significantly higher in proliferative lesions (Pro III) compared with non-proliferative AKs (Pro I), with a higher proportion of Pro III cases showing 1–10% and 11–50% podoplanin-positive cells (*p* < 0.0001, *p* = 0.0039). (**c**) Assessment of podoplanin expression based on the semi-quantitative immunoreactive score (IRS) according to Remmele and Stegner [11,12]. The IRS equals the percentage of positive cells (0–4) multiplied by staining intensity (0–3), resulting in a score from 0 to 12 (0 = no expression, 1–2 = weak, 3–4 = moderate, 6–12 = strong). Weak expression was significantly more frequent in the Pro III group, while 42.9% of AKs in the Pro I group showed no detectable podoplanin expression.

Immunohistochemically, no differences were found in the expression level and distribution of Ki-67 between Pro I and Pro III (*p* = 0.3588). The majority showed weak expression (54.6%), followed by moderate (36.1%) and, less frequently, strong expression (8.3%). Positivity was predominantly limited to the lower third of the epidermis (55.7%). Overall, p53 expression was high (88.7%) and comparable in both groups, although strong staining intensity occurred more frequently in Pro III (25.0% vs. 6.1%; *p* = 0.0105). The distribution of positive cells in the epidermal layers did not differ significantly between Pro I and Pro III (*p* = 0.3476). For p16, no differences were observed in expression level or staining intensity between Pro I and Pro III (*p* = 0.2601; *p* = 0.5952). Lack of expression predominated in both groups (55.7%), while distribution in the lower two-thirds of the epidermis was significantly more frequent in Pro III (22.8% vs. 8.2%; *p* = 0.0456). Podoplanin was significantly more often undetectable in Pro I (42.9% vs. 6.3%; *p* < 0.0001), while proportions of 1–10% (45.8% vs. 18.4%; *p* = 0.0017) and 51–80% (8.3% vs. 0%; *p* = 0.0401) positive cells predominated in Pro III. In Pro III lesions, weak staining intensity (66.7% vs. 34.7%; *p* = 0.0017), a low IRS (66.6% vs. 36.7%; *p* = 0.0033) and localization of positive cells in the lower epidermal third (87.5% vs. 57.1%; *p* = 0.0009) occurred more frequently. The distribution of histological grades and podoplanin immunoreactivity is shown in Figure 1. Detailed immunohistochemical findings are summarized in Table 2. 

## 4. Discussion

Previous classification systems for AKs, such as the clinical classification by Olsen et al. [13], the histological grading by Röwert-Huber et al. [7], or the PRO classification by Schmitz et al. [9], enable systematic categorization based on clinical and histological criteria but are not sufficient to clearly distinguish high-risk from low-risk AKs.

The aim of this study was therefore to investigate immunohistochemical markers (Ki-67, p53, p16, podoplanin) in relation to basal proliferation patterns, in order to identify markers associated with distinct basal proliferation patterns and histopathological features that may be linked to the potential risk of progression to cSCC. Our results showed a significant correlation between histological classification and basal growth pattern. Pro III lesions were significantly more often classified as AK III (47.9% vs. 14.3% in Pro I), while AK I predominantly occurred in PRO I (40.8% vs. 18.8% in Pro III). In addition, acantholysis occurred significantly more often in Pro III AKs (22.9% vs. 0%; *p* = 0.0004). These findings confirm previous studies showing that the PRO III growth pattern is strongly associated with higher-grade AKs and is more frequently detected in the immediate vicinity of invasive SCCs [14].

Acantholysis refers to the loss of desmosomal connections between keratinocytes, leading to reduced intercellular cohesion and the formation of intraepidermal clefts [15]. In actinic keratoses, this phenomenon is associated with UV-induced alterations in cell adhesion and impaired DNA repair and may thereby contribute to dysplastic transformation [16,17]. In our cohort, acantholysis correlated significantly with PRO III pattern, although data regarding the progression risk of acantholytic AKs are still limited. A recent study by Falkenberg et al. demonstrated that PRO III combined with acantholysis in AKs may represent a high-risk pattern for iSCC [18]. At the same time, recent reviews indicate that histological features such as atypical keratinocytes in the basal epidermal third and basal proliferative behavior are crucial in determining which AKs may potentially progress malignantly [19].

Among the immunohistochemical markers investigated, clear differences in interpretative value were found. Ki-67 expression was high in both groups but without significant differences. Positive cells were mainly located in basal and parabasal layers, which corresponds to the known staining pattern in AK and explains why Ki-67 cannot differentiate between low- and high-risk AKs. Ki-67 mainly reflects general cell proliferation, regardless of the lesion’s biological aggressiveness [20,21,22,23].

P53 was detected in the majority of AKs (88.7%) without differences between proliferative and non-proliferative groups. This suggests that p53 mutations represent an early and frequent event in AK development but do not provide additional information for risk stratification. In the literature, data on p53 are inconsistent. While some studies reported an association between p53 overexpression and keratinocyte atypia or progression [16,24], other studies suggested a higher risk in lesions with reduced p53 immunoreactivity [25]. In our cohort, no clear correlation was observed, indicating that p53 is not suitable for further differentiation of AK subgroups.

Similarly, p16 was detectable in 44.3% of cases, usually with weak to moderate expression and predominantly basal localization, without significant differences between the groups. Previous studies showed stronger expression in Bowen’s disease and SCCs [21,26]. Hodges et al. reported that p16 overexpression increases during AK progression to iSCC but is not sufficient alone, implying that additional factors are involved [27]. Thus, p16 appears to have no independent prognostic significance at the AK stage, though it may gain importance in later tumor development.

**Figure 2:**
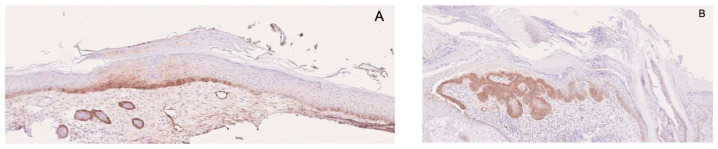
(**A**) Podoplanin expression in AK PRO I with strong staining intensity limited to the basal cell layer (original magnification ×20); (**B**) Podoplanin expression in AK PRO III with distribution in the proliferative areas of the lesion (original magnification ×20).

In contrast to Ki-67, p53 and p16, which showed no differentiating power in our cohort, podoplanin proved to be a clearly discriminative marker. Overall, 75.3% of all AKs were podoplanin-positive, but expression was absent in 42.9% of non-proliferative AKs, compared with only 6.3% of Pro III lesions (*p* < 0.00001). Moreover, PRO III AKs exhibited markedly stronger expression, both in positivity rate and staining intensity, with predominantly basal localization (*p* < 0.05). Representative podoplanin staining patterns in PRO I and PRO III lesions are shown in Figure 2.

These observations are of particular biological relevance, as podoplanin plays a functional role in cell migration, invasiveness and epithelial–mesenchymal transition (EMT) across various tumors [11,28,29].

In differentiated cutaneous SCCs, podoplanin overexpression has been associated with deeper infiltration and higher metastatic potential [11,29]. The fact that podoplanin is already expressed at higher levels in proliferative AKs with PRO III patterns suggests that these lesions activate molecular invasion programs at an early stage. This suggests that podoplanin may act not only as a correlate but also as a potential driver of progression. Nevertheless, definitions and scoring criteria for podoplanin positivity vary in the literature, making direct inter-study comparisons difficult.

An experimental study on cSCC suggests that podoplanin primarily influences the invasive properties of tumor cells. When podoplanin was knocked out in murine cSCC cells using CRISPR/Cas9, the cells showed reduced migration and invasion. Moreover, smaller tumors with less stromal infiltration developed in the orthotopic mouse model. Since Ki-67 expression remained unchanged, these findings suggest that podoplanin affects tumor cell invasiveness rather than proliferation [28].

Notably, the combination of podoplanin expression and PRO III defines a distinct histopathological profile. Podoplanin-positive PRO III AKs show biological and histopathological features that may be relevant to progression toward iSCC, whereas podoplanin-negative PRO I AKs showed fewer of these features in our cohort. Therefore, podoplanin could complement existing histopathological classifications and support future risk-adapted therapeutic decisions. Clinically, this is particularly relevant in the head and neck region, where SCCs carry a higher metastatic risk.

In conclusion, our results indicate that AKs with PRO III, AK III, and acantholysis represent a morphologically and biologically distinct subgroup. Among the markers examined, podoplanin proved the most informative parameter, whereas Ki-67, p53 and p16 offered additional discriminatory value in this cohort. Podoplanin, together with morphological criteria, could form the basis for future AK risk stratification. However, a major limitation of this study is its retrospective design without longitudinal clinical follow-up; prospective, multicenter studies should also assess whether podoplanin can be incorporated into a combined clinical–histological score to identify and treat high-risk patients more precisely.

## 5. Conclusions

Podoplanin expression, particularly in combination with PRO III and acantholysis, identifies a distinct histopathological subgroup of actinic keratoses. In this cohort, podoplanin was more strongly associated with proliferative lesions than Ki-67, p53, or p16. These findings suggest that podoplanin may complement existing classification systems, but prospective longitudinal studies are required before any prognostic significance for progression to invasive squamous cell carcinoma can be established.

## 6. Limitations of the Study

Our study was limited by its single-center, retrospective design and the relatively small number of AKs (n = 97). We included only lesions from the face and scalp in order to reduce heterogeneity, which may have influenced the results and limited the representativeness of the overall AK spectrum. The exclusion of lesions with a PRO II growth pattern represents a further limitation of this study, as it may limit the generalizability of the findings and introduce a degree of selection bias. Differences in patient age and anatomical site distribution between PRO I and PRO III lesions may also have affected the observed associations. In addition, thresholds for classifying podoplanin expression as positive or negative are not standardized, and their sensitivity and specificity for prognostic or risk-stratification purposes have not yet been established. Moreover, the absence of longitudinal follow-up data prevents us from drawing conclusions regarding the true prognostic value of the investigated markers for progression to invasive cSCC. To gain a more comprehensive understanding of this topic, larger prospective multicenter studies with longitudinal follow-up are necessary.

## Figures and Tables

**Table 1:** Demographic and histological characteristics.

Characteristics	Overall (*N* = 97)	Proliferative AK (Pro III) (*N* = 48)	Non-Proliferative AK (Pro I) (*N* = 49)	*p*-Value
	*n* (%)	*n* (%)	*n* (%)	(Pro I vs. { XE “vs.” \t “versus” }Pro III)
Demography				
Sex ^#^				0.0207 *
Male	66 (68%)	38 (79.2%)	28 (57%)	
Female	31 (32%)	10 (20.8%)	21 (43%)	
Age, years ^#^	77 (68–82) ^a^	78 (69.75–84) ^a^	73 (64–79) ^a^	
Location of lesion				
Bald scalp ^#^	26 (26.8%)	7 (14.6%)	19 (38.8%)	0.0075 *
Forehead ^#^	28 (28.9%)	19 (39.6%)	9 (18.4%)	0.0218 *
Left side of the face (+ear)	22 (22.7%)	12 (25%)	10 (20.4%)	0.5912
Right side of the face (+ear)	21 (21.6%)	10 (20.8%)	11 (22.4%)	0.8476
Histological Characteristics				
Histological Severity ^#^				0.0005 *
AK I ^#^	29 (29.9%)	9 (18.8%)	20 (40.8%)	0.0182 *
AK II	38 (39.2%)	16 (33.3%)	22 (44.9%)	
AK III ^#^	30 (30.9%)	23 (47.9%)	7 (14.3%)	0.0004 *
Parakeratosis				0.0588
Mild	69 (71.2%)	30 (62.4%)	39 (79.6%)	
Moderate	24 (24.7%)	15 (31.3%)	9 (18.4%)	
Severe	4 (4.1%)	3 (6.3%)	1 (2%)	
Elastosis				0.2600
Mild	29 (29.9%)	12 (25%)	17 (34.7%)	
Moderate	65 (67%)	34 (70.8%)	31 (63.3%)	
Severe	3 (3.1%)	2 (4.2%)	1 (2%)	
Infiltrate ^#^				<0.0001 *
None ^#^	25 (25.8%)	3 (6.3%)	22 (44.9%)	<0.0001 *
Mild	52 (53.6%)	28 (58.3%)	24 (49%)	
Moderate ^#^	17 (17.5%)	14 (29.2%)	3 (6.1%)	0.0030 *
Severe	3 (3.1%)	3 (6.3%)	0 (0%)	
Acantholysis ^#^				0.0004 *
Yes ^#^	11 (11.3%)	11 (22.9%)	0 (0%)	
No ^#^	86 (88.7%)	37 (77.1%)	49 (100%)	

AK: actinic keratosis; ^a^ Data are median values (interquartile range [IQR]); ^#^ these characteristics differ significantly (*p* < 0.05) between the two groups (proliferative AK [Pro III] and non-proliferative AK [Pro I]); * these values are statistically significant.

**Table 2:** Immunohistochemical characteristics of proliferative (Pro III) and non-proliferative (Pro I) actinic keratoses.

Characteristics		Overall (*N* = 97)	Proliferative AK (Pro III) (*N* = 48)	Non-Proliferative AK (Pro I) (*N* = 49)	*p*-Value
		*n* (%)	*n* (%)	*n* (%)	(Pro I vs. { XE “vs.” \t “versus” } Pro III)
Ki-67 Immunoreactivity					
Percentage of positive cells					0.3588
No expression	<1%	1 (1%)	1 (2.1%)	0 (0%)	
Weak	1–20%	53 (54.6%)	28 (58.3%)	25 (51%)	
Moderate	21–40%	35 (36.1%)	15 (31.3%)	20 (40.8%)	
Strong	>41%	8 (8.3%)	4 (8.3%)	4 (8.2%)	
Ki-67 Localization in the epidermis					0.1833
Lower third (AK I)		54 (55.7%)	24 (50%)	30 (61.2%)	
Lower two-thirds (AK II)		40 (41.2%)	21 (43.8%)	19 (38.8%)	
Entire Epidermis (AK III)		2 (2.1%)	2 (4.2%)	0 (0%)	
Not assessable		1 (1%)	1 (2%)	0 (0%)	
P53 Immunoreactivity					
Percentage of positive cells					0.4683
No expression.	<1%	11 (11.3%)	4 (8.3%)	7 (14.3%)	
Weak	1–25%	36 (37.1%)	19 (39.6%)	17 (34.7%)	
Moderate	26–50%	14 (14.5%)	5 (10.4%)	9 (18.4%)	
Strong	>51%	36 (37.1%)	20 (41.7%)	16 (32.6%)	
P53 staining intensity					0.4409
None		11 (11.3%)	4 (8.3%)	7 (14.3%)	
Weak		39 (40.2%)	18 (37.5%)	21 (42.9%)	
Moderate		32 (33%)	14 (29.2%)	18 (36.7%)	
Strong *^#^*		15 (15.5%)	12 (25%)	3 (6.1%)	0.0105 *
P53 Localization in the epidermis					0.3476
Lower third (AK I)		27(27.9%)	16 (33.3%)	11 (22.4%)	
Lower two-thirds (AK II)		43 (44.3%)	19 (39.6%)	24 (49%)	
Entire Epidermis (AK III)		16 (16.5%)	9 (18.8%)	7 (14.3%)	
Not assessable		11 (11.3%)	4 (8.3%)	7 (14.3%)	
P53 positive cells					0.6126
Not only in pro zones		60 (61.9%)	27 (56.3%)	33 (67.3%)	
Only in proliferative zones		26 (26.8%)	17 (35.4%)	9 (18.4%)	
Not assessable		11 (11.3%)	4 (8.3%)	7 (14.3%)	
Podoplanin-Immunoreactivity					
Podoplanin Immunoreactivity		73 (75.3%)	45 (93.75%)	28 (57.1%)	
Percentage of positive cells *^#^*					0.0056 *
No positive cells ^#^		24 (24.7%)	3 (6.3%)	21 (42.9%)	<0.0001 *
1–10% *^#^*		31 (32%)	22 (45.8%)	9 (18.4%)	0.0039 *
11–50%		38 (39.2%)	19 (39.6%)	19 (38.8%)	
51–80% *^#^*		4 (4.1%)	4 (8.3%)	0 (0%)	0.0401 *
>81%		0 (0%)	0 (0%)	0 (0%)	
Podoplanin staining intensity *^#^*					0.0029 *
None *^#^*		24 (24.7%)	3 (6.3%)	21 (42.9%)	<0.0001 *
Weak *^#^*		49 (50.5%)	32 (66.7%)	17 (34.7%)	0.0017 *
Moderate		19 (19.6%)	9 (18.8%)	10 (20.4%)	
Strong		5 (5.2%)	4 (8.3%)	1 (2%)	
Remmele and Stegner Score *^#^*					0.0016 *
No expression *^#^*		24 (24.7%)	3 (6.3%)	21 (42.9%)	<0.0001 *
Weak *^#^*		50 (51.5%)	32 (66.6%)	18 (36.7%)	0.0033 *
Moderate		18 (18.6%)	9 (18.8%)	9 (18.4%)	
Strong		5 (5.2%)	4 (8.3%)	1 (2%)	
Podoplanin Localization in the epidermis ^#^					0.0003 *
Lower third (AK I)		70 (72.2%)	42 (87.5%)	28 (57.1%)	0.0009 *
Lower two-thirds (AK II)		2 (2.1%)	2 (4.2%)	0 (0%)	
Entire Epidermis (AK III)		1 (1%)	1 (2%)	0 (0%)	
Not assessable		24 (24.7%)	3 (6.3%)	21 (42.9%)	
P16 Immunoreactivity					
P16 Positivity					0.2601
No expression	<1%	54 (55.7%)	25 (52.1%)	29 (59.2%)	
Weak to moderate	1–30%	31 (32%)	14 (29.2%)	17 (34.7%)	
Strong	>31%	12 (12.3%)	9 (18.8%)	3 (6.1%)	
P16 staining intensity					0.5952
None		54 (55.7%)	25 (52.1%)	29 (59.2%)	
Weak		15 (15.5%)	8 (16.6%)	7 (14.3%)	
Moderate		19 (19.6%)	9 (18.8%)	10 (20.4%)	
Strong		9 (9.2%)	6 (12.5%)	3 (6.1%)	
P16 Localization					0.0034 *
Lower third (AK I)		25 (25.8%)	9 (18.8%)	16 (32.6%)	
Lower two-thirds (AK II)		15 (15.5%)	11 (22.8%)	4 (8.2%)	0.0456 *
Entire Epidermis (AK III)		3 (3%)	3 (6.3%)	0 (0%)	
Not assessable		54 (55.7%)	25 (52.1%)	29 (59.2%)	

AK: actinic keratosis; ^#^ these characteristics differ significantly (*p* < 0.05) between the two groups (proliferative AK [Pro III] and non-proliferative AK [Pro I]); * these values are statistically significant. Remmele and Stegner scoring = percentage of positive cells (0–4) × intensity of staining (0–3) (0 points = no expression, 1–2 points = weak expression, 3–4 points = moderate expression, 6–12 points = strong expression) [11,12].

## Data Availability

The data presented in this study are available on request from the corresponding author.
